# Supplementary material for: Design of a comprehensive microfluidic and microscopic toolbox for the ultra-wide spatio-temporal study of plant protoplasts development and physiology
Source: Plant Methods. 2019 Jul 24;15:79. doi: 10.1186/s13007-019-0459-z (PMC6651895; doi:10.1186/s13007-019-0459-z)
Supplement: Supplementary file 1 — Additional file 1. Supplementary informations and detailed methods. [file 13007_2019_459_MOESM1_ESM.docx]

**SUPPLEMENTARY INFORMATIONS**

**Design of a microfluidic and microscopic toolbox for the ultra-wide spatio-temporal study of plant protoplast development and physiology**

K. Sakai^1,2^, F. Charlot^3, 4^ , T. Le Saux^1^, S. Bonhomme^3, 4^, F. Nogué^3, 4^, J.C. Palauqui*^3, 4^, J. Fattaccioli*^1,2^

^1^ PASTEUR, Département de Chimie, École Normale Supérieure, PSL University, Sorbonne Université, CNRS, 75005 Paris, France

^2^ Institut Pierre-Gilles de Gennes pour la Microfluidique, 75005 Paris, France

^3^ INRA, Institut Jean-Pierre Bourgin, Saclay Plant Sciences, Versailles, France

^4^ AgroParisTech, Institut Jean-Pierre Bourgin, Saclay Plant Sciences, Versailles, France

*Corresponding author(s) e-mail: jean-christophe.palauqui@inra.fr and jacques.fattaccioli@ens.fr

[1. Effect of an overnight laser scanning confocal microscopy observation on growth 2](#_Toc12208929)

[2. Evolution of the temperature in the experimental chamber over time 2](#_Toc12208930)

[3. Immobilization and growth of *Physcomitrella patens* spores. 3](#_Toc12208931)

[4. Preparation of solutions and culture media 4](#_Toc12208932)

[5. Preparation of solid culture media 7](#_Toc12208933)

[6. Fabrication of the microfluidic chips 7](#_Toc12208934)

[7. Protonema amplification and protoplasts isolation 11](#_Toc12208935)

[8. Protonema immobilization and culturing in microfluidic devices 11](#_Toc12208936)

[9. References 13](#_Toc12208937)

# Effect of an overnight laser scanning confocal microscopy observation on growth


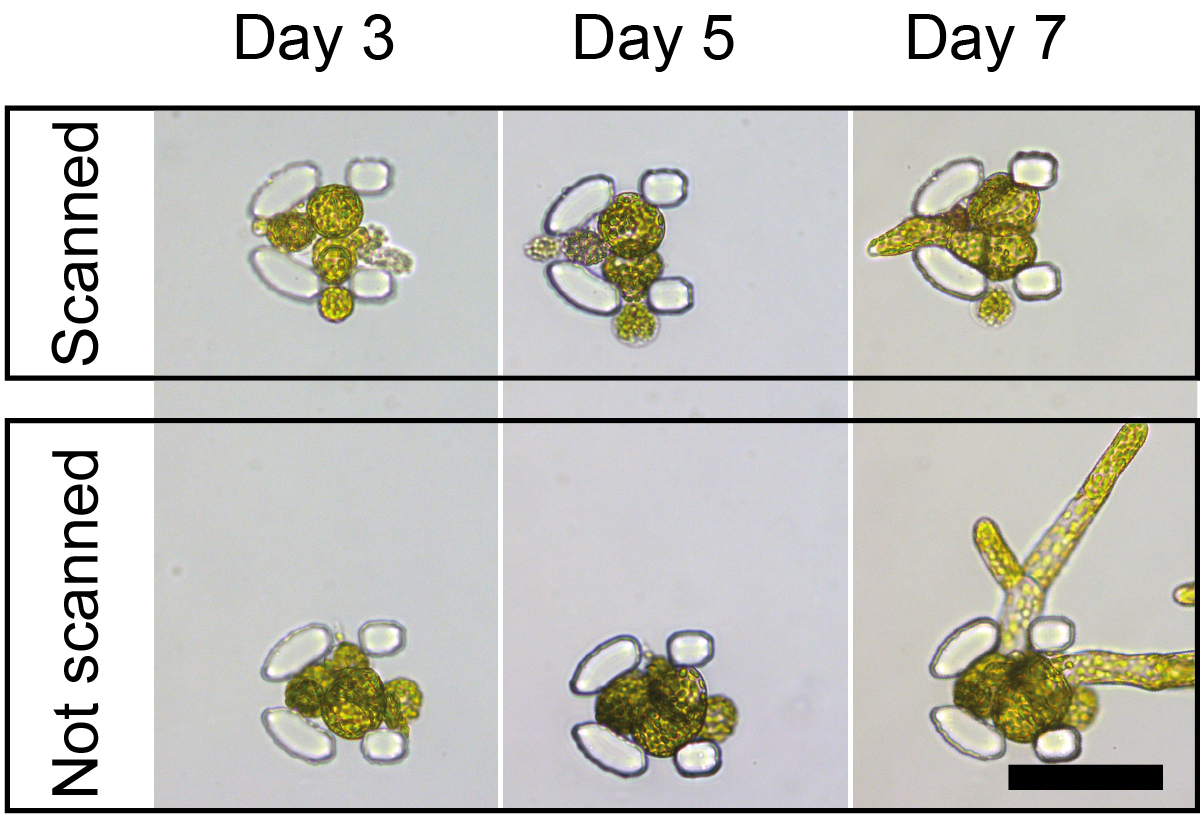


**Figure S 1:** Brightfield time-lapse imaging and evolution of two adjacent traps containing protoplasts at day 3 after loading in the microchip, having (top) or not (bottom) experienced an overnight 5D laser scanning confocal microscopy session. Images recorded right after (**Day 3**), 48 h after (**Day 5**) and 96 h after (**Day 7**) the confocal microscopy session. While immobilized protoplasts having experienced confocal microscopy imaging don’t grow, protoplasts which haven’t been scanned develop normally. Scale : 50 µm

# Evolution of the temperature in the experimental chamber over time

**Figure S 2:** Evolution of the temperature (°C, red) in the chamber containing the in-house microscope, during a full photoperiod (blue). The light intensity is expressed in arbitrary units.

# Immobilization and growth of *Physcomitrella patens* spores.


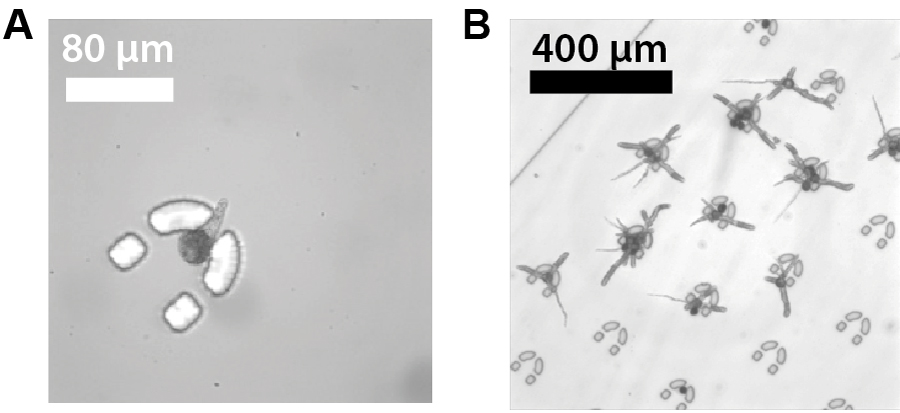


**Figure S 3:** Development of *P. patens* spores immobilized in the microfluidic trap and fed continuously with a PpNH4 medium. (A) Germination takes places 3 days after immobilization. (D) 5 days after trapping, most of the spores have germinated and started to develop.

# Preparation of solutions and culture media

## Preparation of the stock solutions

### Stock solution A (100x, Macro elements)

|  | CAS# | Quantity | Final concentration (mol/L) |
| --- | --- | --- | --- |
| DI water |  | 100m L |  |
| Ca(NO_3_)_2_ 4H_2_O | 13477-34-4 | 0.8 g | 3.39E-02 (MW=236.15 g/mol) |
| MgSO_4_ 7H_2_O | 10034-99-8 | 0.25 g | 1.01E-02 (MW=246.47 g/mol) |
| FeSO_4_ 7H_2_O | 7782-63-0 | 0.0125 g | 4.50E-02 (MW=278.01 g/mol) |

1. Dissolve the above ingredients in a graduated flask with a magnetic stirrer
2. Store the solution at 4°C

### Stock solution B (Microelements, 1000x, stored at 4°C) :

|  | CAS# | Quantity | Final concentration (mol/L) |
| --- | --- | --- | --- |
| DI water |  | 100 mL |  |
| CuSO_4_ 5H_2_O | 7758-99-8 | 5.5 mg | 2.20E-04 (MW=249.7 g/mol) |
| ZnSO_4_ 7H_2_O | 7446-20-0 | 5.5 mg | 1.91E-04 (287.56 g/mol) |
| H_3_BO_3_ | 10043-35-3 | 61.4 mg | 9.93E-03 (61.83 g/mol) |
| MnCl_2_ 4H_2_O | 13446-34-9 | 38.9 mg | 1.97E-03 (197.91 g/mol) |
| CoCl_2_ 6H_2_O | 7791-13-1 | 5.5 mg | 2.31E-04 (237.93 g/mol) |
| KI | 7681-11-0 | 2.8 mg | 1.69E-04 (166.00 g/mol) |
| Na_2_MoO_4_ 2H_2_O | 10102-40-6 | 2.5 mg | 1.03E-04 (241.95 g/mol) |

1. Dissolve the above ingredients in a graduated flask with a magnetic stirrer
2. Store the solution at 4°C

### Stock solution C (KOH, 10N, stored at room temperature)

|  | CAS# | Quantity | Final concentration (mol/L) |
| --- | --- | --- | --- |
| DI water |  | 50 mL |  |
| KOH | 1310-58-3 | 28g | 9.98 (MW=56,11 g/mol) |

1. Dissolve the above ingredients in a graduated flask with a magnetic stirrer
2. Store the solution at room temperature

### Stock solution D (Phosphate buffer, 1000X)

|  | CAS# | Quantity | Final concentration (mol/L) |
| --- | --- | --- | --- |
| DI water |  | 100 mL |  |
| KH_2_PO_4_ | 7778-77-0 | 25g | 1.84 (MW=136.09 g/mol) |

1. Dissolve the above ingredients in a graduated flask with a magnetic stirrer
2. Adjust the pH to pH=7 by adding Stock solution C dropwise under constant monitoring with a pH-meter
3. Store the solution at 4°C

## Preparation of liquid culture media and solutions

### Liquid PpNH4 medium (Ashton, 1979)

| Ingredient | CAS # | Quantity |
| --- | --- | --- |
| DI water |  | q.s. 1L |
| Stock solution A |  | 10 mL |
| Stock solution B |  | 1 mL |
| Stock solution C |  | 1 mL |
| Ammonium tartrate | 3164-29-2 (mw=184.15 g/mol) | 500 mg |

1. Dissolve and mix the above ingredients in a graduated flask with a magnetic stirrer
2. Autoclave the solution to make it sterile
3. Store the solution at room temperature after autoclaving

### 8.5 wt% d-mannitol solution

| Ingredient | CAS # | Quantity | Final concentration | Final concentration (mol/L) |
| --- | --- | --- | --- | --- |
| DI water |  | 1 L |  |  |
| d-mannitol | 69-65-8 | 85 g | 8.5wt% | 4.67E-01  (MW=182.17) |

1. Dissolve and mix the above ingredients in a graduated flask with a magnetic stirrer
2. Autoclave the solution to make it sterile
3. Store the solution at room temperature after autoclaving

### Iso-osmotic medium

| Ingredient | CAS # | Quantity | Final concentration | Final concentration (mol/L) |
| --- | --- | --- | --- | --- |
| Stock solution A |  | 1 L |  |  |
| d-mannitol | 69-65-8 | 66 g | 6.6wt% | 3.62E-01 |
| d-glucose | 14431-43-7 | 5 g | 0.5wt% | 2.52E-02  (198.17 g/mol) |

1. Dissolve and mix the above ingredients in a graduated flask with a magnetic stirrer
2. Autoclave the solution to make it sterile
3. Store the solution at room temperature after autoclaving

### Intermediate medium

| **Ingredient** | **CAS #** | **Quantity** | **Final concentration** | **Final conc. (mol/L)** |
| --- | --- | --- | --- | --- |
| Stock solution A |  | 1 L |  |  |
| d-mannitol | 69-65-8 | 33 g | 3.3wt% | 1.81E-01 |
| d-glucose | 14431-43-7 | 2.5 g | 0.25wt% | 1.26E-02 |

1. Dissolve and mix the above ingredients in a graduated flask with a magnetic stirrer
2. Autoclave the solution to sterilize it
3. Store the solution at room temperature after autoclaving

## 2 wt% driselase solution

| **Ingredient** | **CAS #** | **Quantity** | **Final concentration** | **Final conc. (mol/L)** |
| --- | --- | --- | --- | --- |
| **DI water** |  | 100 mL |  |  |
| **Driselase** | 85186-71-6 | 2 g | 2 wt% |  |
| **d-mannitol** | 69-65-8 | 8.5 g | 8.5 wt% | 4.67E-1 |

1. Dissolve and mix the above ingredients in a graduated flask with a magnetic stirrer for 30min
2. Centrifuge 10min at 2500g
3. Filter sterilize with 0,2µm filter
4. Aliquot 10 mL into sterile plastic tube
5. Store in a freezer at –20°C

# Preparation of solid culture media

## Solid PpNH4 medium (autoclaved and stored at RT):

| Ingredient | REF |  |
| --- | --- | --- |
| Liquid PpNH4 |  | 1 L |
| Agar | VITRO AGAR-1 (Kalys) | 7.2 g |

1. Dissolve and mix the above ingredients in a graduated flask with a magnetic stirrer
2. Autoclave the solution to make it sterile
3. Store the solution at room temperature after autoclaving

# Fabrication of the microfluidic chips

## Mold fabrication

The SU8 on silicon mold fabrication procedure is commonly called *soft lithography* [1]*.*

### Consumables and instruments

1. Silicon wafer (Orientation: 100, Thickness : 475+/-25µm, , Supplier).
2. Epoxy-based negative photoresist (SU-8 2050, MicroChem).
3. Developer solution : SU-8 developer (Microchem) or PGMEA (ULSI grade, Technic Inc.).
4. Negative chromium mask or plastic laser printed mask: Selba (Selba.ch) or Photomask Co (Photomask.co.uk). Minimal features on the design demand for a relatively high printing resolution (5 µm or less).
5. High-grade isopropanol (GPR Rectapur, Propan-2-ol, VWR).
6. Crystallizing pan.
7. Two hotplates (Super-Nuova, Thermo scientific).
8. Spincoater (LabSpin, Süss MicroTec).
9. Mask aligner (Karl-Süss MJB4 or Kloé UV-KUB series).
10. Petri dishes (145x20mm, ref : 391-3665, Greiner Bio-One)

### Procedure

Before starting the procedure, we advise you to

- read carefully the Microchem SU-8 datasheet to have all details of the process in mind.
- place photoresist and all chemicals at room temperature at least 30 min before experiments to avoid humidity condensation.

1. Bake the coated wafer for 15 min at 200°C on a hotplate to evaporate water molecules from the wafer surface and improve the photoresist adhesion.
2. Place the wafer on the spin-coater and pour an adapted volume of photoresist in the center. For a 4 inches wafer, a 5-10 mL volume is sufficient.
3. Typical spincoating parameters for photoresist are 500 rpm for 5 s (acceleration of 100 rpm/s) and 30 s at a speed proportional to the thickness wanted (e.g., 50 um with SU-8 2050 requires a final speed of 3,000 rpm, see application notes given by the supplier).
4. Softbake step: place the wafer on the hotplate at 65°C for 2 min and then for 8 min at 95°C.
5. Place the mask on the baked layer of photoresist. The mask must be very clean in order to ensure a good contact between the mask and the photoresist. Use an air gun to remove dust. Press the mask against the wafer to ensure a good contact. If no vacuum holder is available, you can place a weight on the mask.
6. Insolate the photoresist through the mask according to the thickness of the layer. For example, for 5 mm, photoresist needs 50 mJ/cm² to be fully activated, while a 30-mm-thick layer requires 150 mJ/cm². This step is the most important one as an over- or an underexposure will strongly affect the shape of the channels. It is common to make a few try on pieces of wafers to calibrate the timing correctly.
7. A postexposure bake (PEB) step ensures the cross-linking of the activated region obtained through illumination. Duration of the bake depends on the thickness of the layer. Put the wafer on a hotplate set at 65°C for 2 min, then on another hotplate set at 95°C (6 min for 50 µm).
8. Once the wafer is back at room temperature, place it in a crystallizing pan containing the developer solution. Agitate gently all along the development process. For 50 µm, this may take up to 10 min.
9. Rinse the wafer with propan-2-ol. If unexposed photoresist remains on the wafer, white traces will appear. Repeat the development and rinsing process until no traces appear. It may be useful to have some development solution in a wash bottle to rinse the wafer when developing very small structures.
10. Hard bake. This step avoids photoresist detachment and relaxes the small cracks that appear during the photolithography process. The wafer is placed on a hotplate at 150°C for 10 min.
11. As vertical dimensions obtained can be hard to assess with a microscope, a mechanical (Dektak 6M, Veeco) or optical interferometer (Wyko NT9100, Veeco) can be used for a precise measurement of features.
12. At the end of the process, insert the wafer in a Petri dish and store it in a shelf if you don’t proceed to PDMS molding right after the lithography process.

## Microfluidic devices fabrication

### Consumables and instruments

1. Curable silicone rubber (e.g., Sylgard 184, Dow Corning and RTV 615, GE Silicones).
2. Silicon/SU-8 mold fabricated following the procedure above
3. Vacuum bell jar (Polycarbonate, Nalgene or Kartell)
4. Vacuum pump or vacuum line
5. 50 mL centrifuge tubes (ex. Corning Falcon)
6. Disposable weighing dishes or plastic cups
7. Aluminium sheets (VWR, ref. 611-9001)
8. Centrifuge with a adaptor for 50 mL centrifuge tubes (e.g. EBA 280, Hettich)
9. Oven with a temperature set at 70°C (e.g. Venti-Line, VWR).

### Procedure

1. Take the silicon mold in its Petri dish
2. Prepare a mix of uncured silicon rubber (A) and curing agent (B) at a weight ratio of 9:1 in a disposable weighing dish or a plastic cup. For a 4” wafer, 45g of A and 5 g of B are sufficient. Mix the two parts thoroughly to insure a homogeneous reaction. Pour the mixture in a 50 mL centrifuge tube and close the tube.
3. Insert the tube in the centrifuge to remove the largest bubbles. 30s at 4000g are sufficient, but you can adjust the time and speed if needed.
4. Pour the PDMS mixture onto the mold in the Petri dish. Thickness of the PDMS should be between 5 mm and 1 cm to insure a strong insertion of the tubing in the chip after glass bonding.
5. Insert the Petri dish with silicon master in a vacuum bell jar for at least 1h, until all the remaining bubbles disappear.
6. When no bubble is visible anymore, place the Petri dish containing the silicon mold and the PDMS in an oven at 70°C for 2h. During curing, the PDMS will reticulate and harden

## Chamber fabrication and assembly

To fabricate the chamber, please use the following routine.

### Consumables and instruments

1. Surgical or razor blade (Single Edge, GEM)
2. Hole driller: core sample cutter (e.g., Rapid-Core Sampling Tool ID=0.5 mm OD = 0.8 mm , 69039-05 , Delta Microscopies)
3. Adhesive tape (3M Transparent Scotch tape)
4. Isopropyl alcohol (Propan-2-ol, GPR Rectapur, Propan-2-ol, VWR)
5. Air or Oxygen Plasma cleaner: PDC 32 G (Harrick Plasma) or Cute Plasma (Femto Science).
6. 35 mm Glass-bottom Petri dishes (WPI Fluorodish P35-100)
7. Cutting mat (Model, Reference, Supplier)
8. Laminar flow hood (Aura vertical S.D.4, BioAir)
9. Air or nitrogen gun or bottle

### Procedure

1. Cut the cured PDMS with a surgical blade, leaving enough space (about 2 mm) around the structures to ease manipulation and allow a good binding to the glass coverslip.
2. Punch holes with the biopsy puncher, from the top part of the PDMS stab to the bottom part where channels are engraved.
3. Clean PDMS chip by sticking and peeling adhesive tape on the structures side.
4. Rinse the bottom side of the PDMS chip with isopropyl-alcohol and dry it carefully with the air or nitrogen gun
5. Proceed to the activation of the surface : place the glass substrate and the PDMS piece with the channels side up in the air/oxygen plasma cleaner. Treat the surfaces at a 20-30W power for 1 min s at a pressure of 500 mTorr.
6. Stick the glass and the PDMS together. Note that surfaces will remain activated only for a short amount of time, so binding should be performed immediately after treatment.
7. Right after having sealed the microchip, flush the chamber with a solution of DI water containing 0.2 wt% Pluronic F127, of an amphiphilic triblock copolymer, and let the solution incubate overnight and up to 2-3 days. During this step, Pluronic F127 coats the surface of PDMS and avoids adhesion of protoplasts to the walls of the chamber.

# Protonema amplification and protoplasts isolation

1. Amplification of protonemal tissue and protoplasts isolation is achieved using standard methods, detailed elsewhere [2–4]
2. -Adjust the concentration of the protoplast suspension to 10^6^ protoplasts per mL . Please insure that the total volume of the suspension is at least of the order of 200-400 µL for easy handling.

# Protonema immobilization and culturing in microfluidic devices

## Reagents

1. Suspension of protoplasts counted above (0,5X10^6^. mL^-1^) in a 8.5 wt% mannitol solution.
2. Liquid media (see section **Preparation of solutions and culture media**)

## Equipment

1. Inverted microscope
2. Pressure regulator (e.g. Fluigent MFCS, range : 0 – 100 kPa or Elveflow OB1)
3. Reservoir holder (e.g. Fluigent Flui-well or Eleveflow 4-XS)
4. Sterile microtubes (Simport T334-6 1.8mL tube)
5. Sample container (60 mL, VWR, ref : 216-2613)
6. Autoclave
7. Tweezers (e.g. RSG Solingen)
8. 0.2 µm sterile filters (Cellulose acetate, diameter : 25 mm, Sartorius)
9. Teflon tubing (ID 0.022mm, OD0.042mm, Cole Parmer)
10. Stainless steel catheters (Size : Gauge 23, 23G-15mm,Phymep, France)

## Immobilization and medium exchange procedure

Proceed to protoplasts loading in the microchip the day of extraction to avoid formation of debris or aggregates, and ultimately make the experiment easier to conduct.

1. Fill a 1.8 mL microtube with the iso-osmotic solution and connect it to the reservoir holder.
2. Prepare the inlet and outlet tubing by inserting one catheter at one end of two pieces of Teflon tubing. The length of the tubing should be sufficient to connect the pressure regulator to the microchip. If the condenser of the microscope is too close from the sample, the catheters can be bent at 90° to avoid the tubing to touch it and make the experiment more difficult to do.
3. Disconnect the catheter at the inlet and connect the catheter corresponding to the iso-osmotic solution. Flush the trapping chamber for 20 min at a pressure of 15 mbar. Check regularly that there is no leakage.
4. Add the protoplast suspension to an empty 1.8 mL microtube,
5. Add the same volume of iso-osmotic solution to the protoplasts suspension
6. Place the microtube to the sample holder and connect the inlet Teflon tubing to the microchip. The outlet tubing connects the chip to a waste container.
7. Under the microscope, increase the inlet pressure slowly until you see protoplasts flowing in the trapping chamber. The pressure should be around 10-15 mbar at the beginning and should be decreased to 5 mbar and maintained for 2-3 min to load individual protoplasts in the microfluidic traps. Important: Check for bubbles that may sit in the device before and after the loading procedure, but also before putting the microfluidic setup in the incubator. Bubbles can be easily removed using a syringe connected to the outlet and by applying a negative pressure that will pump the bubbles out of the chamber.
8. Add DI water in the glass-bottom Petri dish to maintain the humidity of the PDMS in the incubator. The volume should be sufficient to reach half of the height of the PDMS chip.
9. Drill a small hole in the cap of two sample containers
10. Fill one of the sample containers with the first, iso-osmotic, culture medium
11. Insert a catheter at one end of a Teflon tubing, and a 0.2 µm sterile filter at the other end. Sink the filter in the culture medium and connext the tubing to the microchip inlet. Connect the empty sample container to the outlet of the microchip. Adjust the height difference, using spacers as Petri dishes, between the inlet and outlet resevoirs, to ca. 4 cm. This will insure a sufficient hydrostatic pressure to put the media under flow.
12. From D3, when most of the cell divisions occurred, switch the culture medium to the intermediate medium, until D5.
13. From D5, the medium is switched to plain PpNH4 medium indefinitely.


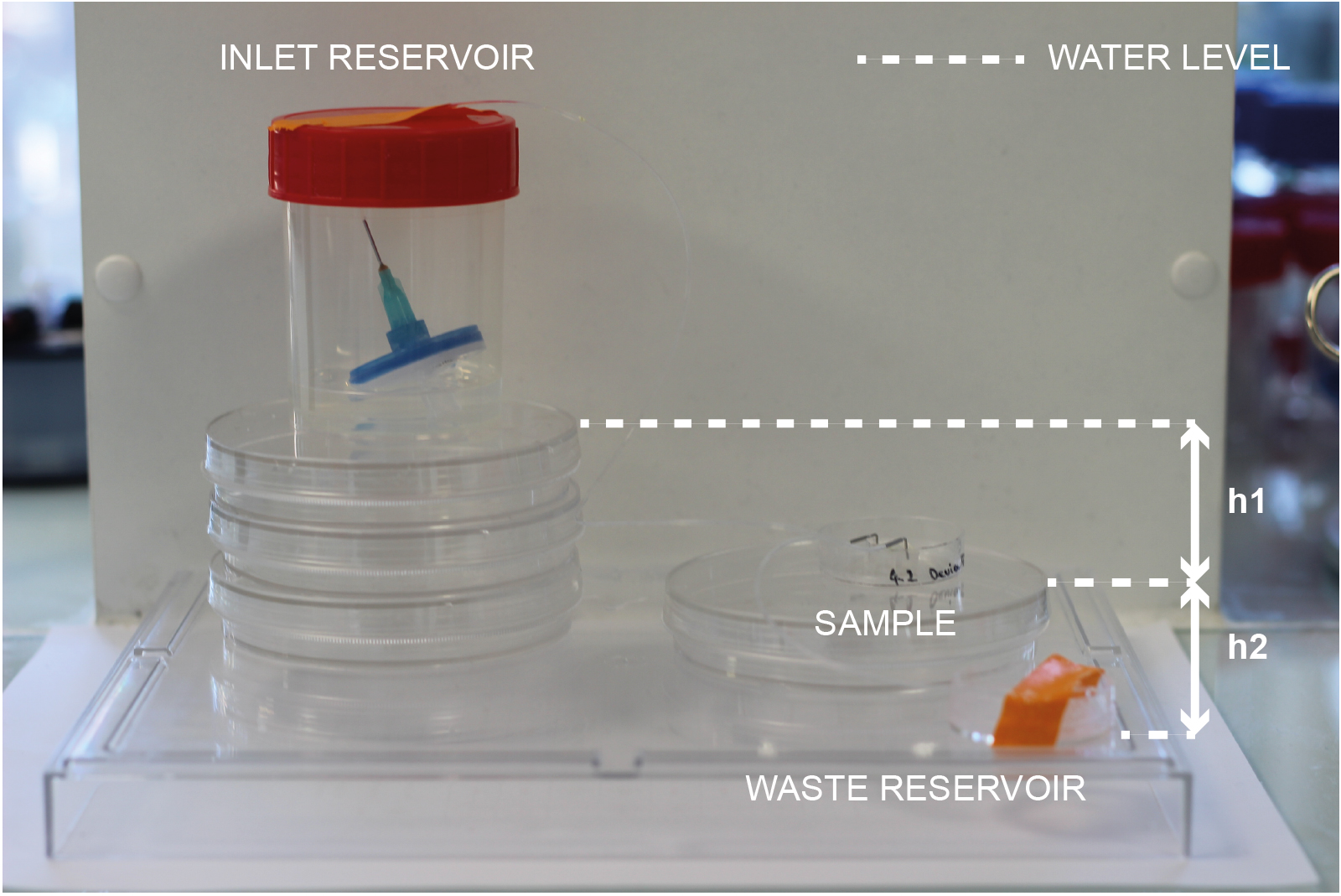


**Figure S 4:** Representative picture of the hydrostatic pressure-controlled fluidic setup. The pressure differences between the inlet reservoir, the chip and the waste reservoir are set by the height differences h1 and h2, respectively.

# References

1. Xia Y, Whitesides GM. Soft Lithography. Angew Chemie Int Ed. 1998;37:550–75.

2. Cove DJ, Perroud P-F, Charron AJ, McDaniel SF, Khandelwal A, Quatrano RS. Culturing the Moss Physcomitrella patens. Cold Spring Harb Protoc. 2009;:doi:10.1101/pdb.prot5136.

3. Cove DJ, Perroud P, Charron AJ, McDaniel SF, Khandelwal A, Quatrano RS. Isolation and Regeneration of Protoplasts of the Moss Physcomitrella patens. Cold Spring Harb Protoc. 2009;:doi:10.1101/pdb.prot5140.

4. Cove DJ, Perroud P-F, Charron AJ, McDaniel SF, Khandelwal A, Quatrano RS. The Moss Physcomitrella patens: A Novel Model System for Plant Development and Genomic Studies. Cold Spring Harb Protoc. 2009;:doi:10.1101/pdb.emo115.
